# Autoimmune thyroid disease and rheumatoid arthritis: where the twain meet

**DOI:** 10.1007/s10067-024-06888-6

**Published:** 2024-02-10

**Authors:** Anna Lichtiger, Golfam Fadaei, Clement E. Tagoe

**Affiliations:** 1 Department of Medicine, Albert Einstein College of Medicine, Bronx, NY USA; 2 Griffin Hospital, Ansonia, CT USA; 3 Division of Rheumatology, Department of Medicine, Albert Einstein College of Medicine, Bronx, NY USA; 4 Division of Rheumatology, Montefiore Medical Center, 111 East 210th Street, Bronx, NY 10467-2490 USA

**Keywords:** Autoimmune thyroid disease, Depression, Disease activity, Fibromyalgia, Osteoarthritis, Rheumatoid arthritis

## Abstract

Autoimmune thyroid disease (AITD) is the most prevalent autoimmune disease. It shares multiple genetic, clinical, and serologic characteristics with rheumatoid arthritis (RA). Although frequently described as a classic form of single-organ autoimmunity, the AITD disease burden in a subset of patients extends well beyond the thyroid gland. This review explores the complex interaction between the two diseases and the clinical consequences when they overlap. Beyond the well-known effects of AITD on thyroid function in RA, there is mounting evidence of the association of both conditions impacting the presentation and outcomes of diabetes, metabolic syndrome, and cardiovascular disease. An increasing number of studies suggest that there are negative effects of AITD on RA disease activity both in the presence and in the absence of thyroid dysfunction. Recent evidence suggests that AITD may not only worsen the cumulative damage of RA through higher disease activity but may also worsen secondary osteoarthritis changes. Less well-known is the significant association between AITD and chronic widespread pain syndromes including fibromyalgia. Importantly, the presence of fibromyalgia, which is increased in RA patients, appears to be further increased when it overlaps with AITD. Lastly, we probe the possible influence of AITD interacting with RA on fertility and clinical depression.

**Table:** 

**Key Points** *• Autoimmune thyroid disease is the most common autoimmune disease and is frequently associated with rheumatoid arthritis.* *• Autoimmune thyroid disease can present with osteoarthritis, inflammatory arthritis, and chronic widespread pain syndromes.* *• The co-occurrence of autoimmune thyroid disease and rheumatoid arthritis may worsen disease activity and exacerbate other disease manifestations including cardiovascular disease, fertility, and depression.* *• The overlap of rheumatoid arthritis with autoimmune thyroid disease needs further research and should be sought in general clinical practice.*

**Key Points**

*• Autoimmune thyroid disease is the most common autoimmune disease and is frequently associated with rheumatoid arthritis.*

*• Autoimmune thyroid disease can present with osteoarthritis, inflammatory arthritis, and chronic widespread pain syndromes.*

*• The co-occurrence of autoimmune thyroid disease and rheumatoid arthritis may worsen disease activity and exacerbate other disease manifestations including cardiovascular disease, fertility, and depression.*

*• The overlap of rheumatoid arthritis with autoimmune thyroid disease needs further research and should be sought in general clinical practice.*

## Introduction

Autoimmune thyroid disease (AITD) is the most prevalent autoimmune disease [1]. It constitutes a spectrum of diseases in which the thyroid gland is invaded by lymphocytes targeting various antigens, leading to a variety of histological changes which produce no clinical phenotype in the majority of patients but can cause hypothyroidism or hyperthyroidism in some cases. The two forms of AITD most commonly described are Hashimoto’s thyroiditis (HT) which is associated with hypothyroidism and Graves’ disease (GD) which is associated with hyperthyroidism. Autoantibodies directed against thyroid antigens are found in the majority of patients with AITD. The anti-thyroid peroxidase antibody (TPOAb) is found in about 11.3% of the general US population and is prevalent in 90–95% of subjects with AITD [2]. When pathogenic it fixes complement and induces antibody-dependent cell-mediated cytotoxicity [3]. The anti-thyroglobulin antibody (TgAb) occurs in about 10.4% of the general US population and is present in 60–80% of those with HT, while the anti-thyroid-stimulating hormone receptor antibody (TSHRAb) is prevalent in over 90% of GD [1, 2]. The occurrence of these autoantibodies indicates the presence of AITD in epidemiologic studies [4]. The best estimates of the population prevalence of hypothyroidism and hyperthyroidism suggest 4.6% and 1.3%, respectively, of the US population [2]. Thus, the majority of those with AITD have normal thyroid function [1].

Rheumatoid arthritis (RA) is a chronic well-defined autoimmune connective tissue disease (ACTD) that is prevalent in about 0.5 to 1% of the US population [5]. It is a systemic disease characterized by symmetric polyarthritis that affects primarily small joints, with symptoms including, morning stiffness, constitutional symptoms, and extraarticular multisystem manifestations such as subcutaneous nodules, vasculitis, pulmonary, cardiovascular, neurologic, and hematologic involvement [6]. There are two associated autoantibodies that aid in the diagnosis of RA, the rheumatoid factor (RF) and the anti-citrullinated protein antibodies (ACPA), the latter being more specific for the disease [7]. Although generally described as single disease entities, autoimmune diseases are frequently found in clusters of polyautoimmunity within patients and are known to share genetic susceptibility [8, 9]. There is considerable enrichment of AITD in patients with RA [10]. A similar increase in the prevalence of AITD is seen in systemic lupus erythematosus, Sjögren’s syndrome, and a variety of other well-defined ACTD [11]. Both conditions affect females more than males, AITD by a factor of 9:1 and RA by a factor of about 3:1 [12, 13]. Recent evidence suggests that AITD has multiple extraglandular associations which can impose a significant disease burden [14, 15]. Here, we review the complex associations between AITD and RA (Table 1), investigating how each may modify the clinical expression of the other and confer risk for several disease states and clinical presentations. We examine how the presence of anti-thyroid antibodies (ATA) might serve as markers for the presence of these other disease states in RA.

**Table 1:** Similarities in genetics, demographics, and clinical presentation of autoimmune thyroid disease and rheumatoid arthritis

Disease characteristics	Autoimmune thyroid disease		Rheumatoid arthritis	Shared features
	Hashimoto’s thyroiditis	Graves’ disease		
Genetics	CTLA-4, PTPN22, HLA-DRB, FOXP3, CD-40, thyroglobulin	TSHR, CTLA-4, CD-40, PTPN22, thyroglobulin, FCRL3, HLA-DRB, IL-2Rα, FOXP3	CTLA-4, PTPN22, FCRL3, HLA-DRB, CD40, FOXP3, TRAF1-C5, PADI4	CTLA-4, PTPN22, HLA-DRB, CD-40, FOXP3
Demographics				
Female predominance	8–9:1	5–10:1	3:1	Female predominance
Age prevalence	Highest at 45–65 years of age	Highest at 30–60 years of age	Highest at 30–50 years of age	Shared age prevalence
Seropositivity	> 90% TPOAb and/or TgAb	> 95% TSHRAb, ~80% TPOAb	~70% RF or ACPA, ~5–35% TPOAb or TgAb	Significant co-occurrence of AITD in RA
Clinical presentation				
Arthritis, chronic widespread pain, fatigue, depression	Common features	Common features	Common features	Considerable overlap in the clinical presentation of RA, HT, and GD

*CTLA-4* cytotoxic T-lymphocyte-associated molecule-4, *FCRL3* Fc receptor-like 3, *FOXP3* forkhead box P3, *GD* Graves’ disease, *HT* Hashimoto’s thyroiditis, *IPEX* immune dysregulation polyendocrinopathy enteropathy X-linked syndrome, *PADI4* peptidylarginine deiminase 4, *PTPN22* protein tyrosine phosphatase nonreceptor type-22, *RA* rheumatoid arthritis, *TRAF1-C5* TNF receptor-associated factor 1-complement component 5, *TSHR* thyrotropin receptor

## Effect of AITD and RA on diabetes, metabolic syndrome, and cardiovascular disease risk

Hypothyroidism confers a significant independent risk of early atherosclerosis, by as much as threefold [16, 17]. The cardiovascular disease (CVD) risk can be partially attributed to hypothyroidism’s association with dyslipidemia [18]. In patients with RA and AITD, the CVD risk is amplified by independent risk from RA because of underlying genetic predisposition as well as an inflammatory state [19]. A few specific human leukocyte antigen (HLA)-DRB1 shared epitope alleles, e.g., HLA-DRB1*0401 and HLA-DRB1*0404, are associated with RA and increased risk of CVD [20, 21]. In addition, the inflammation in RA upregulates cytokines, such as tumor necrosis factor-alpha (TNF-α) and interleukin 1 beta (IL-1β), in affected joints that can impact distant sites, create oxidative stress, endothelial dysfunction, plaque instability, and thrombosis [17]. These inflammatory changes drive atherosclerosis and CVD [22, 23]. Furthermore, patients with RA and AITD are more susceptible to thrombosis (13% vs. 5%, OR = 3) [24]. A retrospective review of RA patients found those with hypothyroidism had twice the odds of having CVD, after adjusting for traditional risk factors [25]. The study also found HT to be significantly associated with CVD with a hazard ratio (HR) of 2.7 [25]. Another prospective cohort study of 358 RA patients demonstrated a fourfold increase in CVD in hypothyroid females compared with euthyroid females after controlling for demographic and health risk factors [17].

Both HT and GD are associated with insulin resistance [26, 27]. Subclinical hypothyroidism is also associated with insulin resistance among RA patients (using the Quantitative Insulin Sensitivity Check Index and Homeostasis Model Assessment for insulin resistance) [28]. Type 1 diabetes (T1D) is associated with AITD. A meta-analysis found the weighted prevalence of TPOAb among patients with T1D to be 18% and TgAb to be 12% (versus 11.3–12.8% and 10.4%, respectively, in the general population) [29]. Patients with certain genetic factors, including HLA, autoimmune regulator (AIRE) gene, protein tyrosine phosphatase non-receptor type 22 (PTPN22), forkhead box P3 (FOXP3) protein, interleukin-2 receptor (IL2RA), and Cytotoxic T-lymphocyte-associated protein 4 (CTLA-4), are more susceptible to developing both AITD and T1D [30]. Environmental exposures are also known to predispose patients to AITD and T1D, including vitamin D deficiency, and exposure to infectious agents, like *Helicobacter pylori* and certain commensal microbes [30]. A cross-sectional study of 800 Colombian RA patients found a sixfold increased prevalence of type 2 diabetes among those with RA and AITD versus those with RA alone [24].

Patients with RA have an increased prevalence of metabolic syndrome (MetS) [31]. A study of 154 consecutive RA patients and 85 controls found that the MetS (defined by the World Health Organization as insulin resistance and two of the following: central obesity, dyslipidemia, or hypertension) occurred in 42% of patients with long-term RA, 31% with early RA versus 11% of controls [31]. Since the thyroid is intimately involved with the regulation of basal metabolic rate, it is logical to surmise that alterations in thyroid function could influence the development of the MetS. One cross-sectional population-based study in China found body mass index (BMI), waist circumference, and blood pressure to be higher among the group positive for TPOAb or TgAb than the antibody-negative group. They found that antibody-positive men had more insulin resistance, and antibody-positive women had higher total cholesterol and LDL compared to antibody-negative groups [32]. Similarly, a meta-analysis found that among women with polycystic ovary syndrome (PCOS), a disease highly associated with the MetS and CVD, 26% had AITD versus 10% of those without PCOS [33]. Conversely, a cross-sectional study of over 4000 Portuguese individuals found that having TPOAb was negatively associated with MetS (OR 0.5) [34]. There were mixed results in the previously described cross-sectional study of 800 Colombian patients, which found that patients with RA and AITD had higher BMI (25.5 vs. 23.9) and were less likely to have hypercholesterolemia (40% vs. 56%) [24].

## AITD in RA and RA in AITD

The similarities in CVD burden between RA and AITD may seem unusual for one disease classically described as systemic and another classically described as prototypically single-organ. However, RA and AITD have been known to be comorbid since the 1960s [35, 36]. Studies of this association have yielded variable results due to true variations based on ethnicity, genetics, and importantly, diagnostic criteria for AITD (from positive ATA to subclinical or overt hyper- or hypothyroidism) [37]. Furthermore, the time along the disease course when the diagnosis of AITD and RA is made may impact the apparent rates of co-occurrence. A Swedish case-control and cohort study of 8090 RA patients and 80,782 population-based controls found the risk of developing AITD among RA patients increased more than 5 years prior to RA diagnosis, peaked in the year prior to diagnosis (OR = 9), and declined after the diagnosis (HR = 0.2) [38].

Various studies have investigated the prevalence estimates of RA and AITD comorbidity. A prospective cohort study of 358 RA patients in the Netherlands found that clinical hypothyroidism was significantly more common among RA patients than in the general population (6.8% vs. 2.7%) [17]. Subclinical hypothyroidism, however, was less common among female RA patients (2.5% vs. 18%, *p* < 0.001), which may be attributed to an inflammatory and accelerating effect of RA on AITD, leading to more overt disease and less subclinical disease [17]. In another study, AITD was more prevalent in RA patients than in patients without RA (16% vs. 9%), though not significantly so [39]. In a controlled prospective survey of consecutive hospital and clinic patients in Montreal, thyroid disease was threefold more common among female RA patients than controls with non-inflammatory rheumatic disease (30% vs. 11%) [40]. The possibility of a complex genetic association between RA and AITD is becoming increasingly evident. Using genome-wide association studies (GWAS) data, Liu et al. found a potential causal effect of genetically predicted RA on autoimmune hyperthyroidism and a bidirectional causal relationship between RA and autoimmune hypothyroidism which was also observed with complementary genetic approaches. However, the GWAS, data although extensive, were derived from European populations, and the results would need to be corroborated in other data sources [41].

ATA has also been shown to be closely associated with RA. As previously noted, the presence of ATA is not necessarily indicative of hormonal dysfunction. In one study of RA patients, 37% had TPOAb and 23% had TgAb, whereas only 2.8% of the patients had overt hypothyroidism [42]. A meta-analysis found TPOAb was twofold as common and TgAb was threefold as common among patients with RA than healthy controls [43]. Bianchi et al. [44] found that among RA patients, 9% had TPOAb and 8% had TgAb, a two-to-four-fold increase of ATA compared to controls. A prospective study in Norway found that the prevalence of TPOAb (17% vs. 6%) and TgAb (11% vs. 3%) was three to fourfold higher among RA patients than the general population of the same area [45].

The clinical presentations of AITD and RA share considerable overlap. Both diseases can manifest with muscle fatigue, cramps, pain, weakness, arthralgias, arthritis, and skin changes [46]. This overlap may make parsing out which symptoms are associated with either autoimmune disease challenging. The presence of inflammatory articular symptoms in patients with AITD may represent the co-occurrence of RA, other rheumatic disease, or clinical symptoms of AITD itself. Undifferentiated inflammatory arthropathy (UIA) is a diagnosis ascribed to patients with rheumatic symptoms who do not yet meet the criteria for a specific rheumatic disease [47]. A cross-sectional study of 92 consecutive Venezuelan patients seen in an Endocrinology Division in a major hospital system with HT and no definitive rheumatic disease found 25% of patients had UIA. Of the patients who did not meet the criteria for UIA, 59% had joint symptoms [47].

The comorbidity of AITD and RA may be due to an individual’s predisposition to autoimmunity as well as shared genetic susceptibilities. Both AITD and RA are more common in patients with a positive family history of autoimmunity and demonstrate higher concordance among monozygotic twins [37]. RA and AITD are both multigenic diseases with numerous genetic loci implicated in their disease susceptibility [48]. HLA-DR genes are associated with antigen presentation, and in particular, HLA-DRB1 is a major susceptibility locus of RA. Arginine at position 74 of the HLA-DRB1 is associated with GD susceptibility [49]. Other genetic susceptibility genes that have been implicated in the pathogenesis of AITD and RA include polymorphisms of PTNP22, a T-cell regulatory protein; Fc receptor-like 3 gene, which encodes a protein involved in the function of T regulatory cells; and STAT4, a transcription factor for cytokines [37, 50].

CTLA-4 is another gene associated with the development of RA and AITD. The CTLA-4 gene encodes the molecule of the same name and downregulates T-cell activation by binding to B7 on antigen-presenting cells. The CTLA-4 gene exon1 A49G polymorphism leads to an amino acid substitution and is associated with susceptibility to numerous autoimmune diseases [37, 51]. The effects of this CTLA-4 polymorphism in susceptibility to AITD and RA, however, have shown inconsistent results depending on the population in question. A study of Slovak patients showed that the frequency of the G allele (OR 2.02) and GG genotype (OR 4.49) of the CTLA-4 A49G gene polymorphism is significantly higher in those with HT and RA than in those with neither disease [51]. Similarly, a study of Chinese patients from Taiwan showed that the G allele and GG genotype conferred an increased risk of RA (RR= 1.39 and 1.72, respectively) [52]. Among individuals from England, the G allele was significantly more frequent in those with early RA (OR 1.35) [48]. However, when controlling for comorbid endocrinopathies, particularly AITD and T1D, the statistically significant relationship was lost [48]. Other studies of British and Spanish RA patients found no significant difference in the frequency of the G allele or the GG genotype [53, 54].

Environmental exposures may also contribute to the development of AITD and RA. Smoking cigarettes increases the risk of development of GD and RA, especially in patients with HLA-DRB1 and ACPA, whereas smoking is protective against the development of HT [37, 55]. Adequate vitamin D intake is protective against the development of RA [37]. The association between AITD and vitamin D is yet inconclusive, but meta-analyses and observational studies seem to point to a similar protective effect of vitamin D against the development of AITD [37, 56]. Similarly, benefits have been found for vitamin D alone or in combination with omega 3 fatty acid supplementation on incident autoimmune disease in the VITAL randomized controlled trial, which included rheumatoid arthritis and AITD in the study [57].

## Effect of AITD on RA disease activity

The pathophysiologic mechanisms in AITD that produce RA-like inflammatory arthritis are unclear but may share common pathways. It has been proposed that synovial lymphocytes may produce ATA which contributes to joint inflammation [58]. Punzi et al. [58] found TPOAb in the synovial fluid of 2 RA patients prior to diagnosis of HT via detection of the antibodies in serum. It has also been proposed that elements of the thyroid hormonal network may live in the synovium among RA patients, even in the presence of normal thyroid function. Pörings et al. [59] studied patients with RA and osteoarthritis (OA) and found higher levels of reverse triiodothyronine (T3), a degradation product, in synovial fluid compared to serum which indicated biodegradation of thyroid hormones in the synovium. They also established the presence of thyroid hormones, thyroid hormone transporters, receptors, and deiodinases in the synovium, indicating a thyroid hormonal network exists in the synovium of these patients [59]. Another study showed that IgG antibodies from GD patients and from RA patients can induce cytokines (including IL-16) not only in their own fibroblasts but in the fibroblasts of patients with other autoimmune disease through the insulin-like growth factor-1 receptor (IGF-1R). Thus, antibodies from GD patients can induce cytokines in RA patient fibroblasts and vice versa [60]. Panchangam et al. [61] hypothesized that ATA may target epitopes outside the thyroid, making other organs vulnerable to autoimmune attack. They conducted a retrospective study of 61 HT patients who underwent total thyroidectomy. They found that post-thyroidectomy TPOAb levels decreased from a mean of 339 to 59 IU/L, and there were major improvements in patients’ associated autoimmune features including RA [61]. These common and overlapping immune activation pathways may help to explain the reported exacerbation of disease activity in patients with an RA and AITD overlap [62, 63].

The literature on the effects of ATA and thyroid disease on RA disease activity is less conclusive than reports on the effects of hypothyroidism. Among hospitalized RA patients, those with ATA were significantly more likely to have radiographic joint damage (68% vs. 42%), high-grade synovitis (63% vs. 36%), plasma cell infiltration of the synovium, positive RF, and significantly higher scores on measures of RA disease activity, including the disease activity score-28 (DAS28) and clinical disease activity index (CDAI) [64, 65]. A couple of studies did not confirm the relationship between ATA and RA outcomes [42, 44]. Of note, both of these studies used individual parameters (morning stiffness, fatigue, joint pain, tender and swollen joint counts, elevated erythrocyte sedimentation rate (ESR) and C-reactive protein (CRP), RF, and anti-CCP) to assess RA disease activity rather than validated disease activity measures. In another study of Polish women that demonstrated an increased prevalence of AITD in RA patients, no difference in disease activity was noted between those with AITD and those without. However, the study was not designed to measure disease activity between groups [39].

The data in patients with thyroid dysfunction, particularly hypothyroidism, has been more clearly shown to be associated with worsening RA disease activity, as measured by DAS28, ESR, CRP, rheumatoid factor, modified health assessment questionnaire (MHAQ), and the visual analog scale for pain [66, 67]. An observational cohort study of 439 RA patients from Denmark found that RA patients with thyroid disorders had more aggressive disease with a worse initial response to RA treatment compared to patients with isolated RA after 4 months [68]. A retrospective study of 26 patients with hypothyroidism and RA in 1982 found that restoring patients to a euthyroid state ameliorated their RA activity. It was suggested to be improved by the resolution of superimposed myxoedematous synovitis at a time when the hormonal influence of hypothyroidism was felt to be the primary pathogenic mechanism of the disease worsening [67]. A more modern interpretation might suggest a complex interaction of hormonal and immunological mechanisms of tissue injury. A cross-sectional study of 350 RA patients, looking at thyroid dysfunction as well as AITD assessed by the presence of ATA, found that thyroid dysfunction, TPOAb positivity, and AITD were significantly more common in RA patients with more severe disease activity measured using the DAS28-ESR. Positive TPOAb was more prevalent (OR 2.93) among patients with severe disease activity compared to those in remission [69].

## Risk of cumulative damage including OA (spine and periphery) in RA with AITD

Autoimmune thyroid disorders are associated with peripheral OA and spinal degenerative disc disease [70, 71]. A study from the Third National Health and Nutrition Examination Survey (NHANES III) demonstrated an association between elevated TPOAb concentrations (> 35 IU/mL) and knee chondrocalcinosis, which is typically associated with OA [72].

Hand OA is of particular interest as the major drivers of the disease are genetic, endocrine, and metabolic factors. Unlike other forms of OA, such as of the knee or hip, mechanical risk factors such as the influence of gravity are less likely to propel the progression of hand OA [73]. In the previous study demonstrating chondrocalcinosis in association with AITD in NHANES III, an association with radiographic, tibiofemoral knee OA was not demonstrated [72]. Therefore, hand OA may be more specific for understanding the association between AITD and OA. One study using an in-hospital patient cohort found that those with hand OA had nearly 5 times the odds of having AITD [74]. In another NHANES III study, symptomatic hand OA was found to be more prevalent in subjects 60 years and older with TPOAb or TgAb than those without [75].

Hand pain is a salient manifestation of several autoimmune diseases including arthritis and chronic widespread pain syndromes [76]. A cross-sectional study of over 4800 adults aged 60 years and over from NHANES III examined hand pain as part of the symptom complex of AITD among patients with ATA. It found that those with higher TPOAb concentration (≥ 72 U/mL) were 1.3 times more likely to have hand pain compared to those without TPOAb [77]. Among those with a TPOAb concentration of ≥ 324 U/mL, 42% had hand pain. Similarly, those with a reported history of thyroid disease were more likely to report hand pain (27% vs 18% of those without thyroid disease). The association exists between hand pain and ATA and thyroid disease, but not between hand pain and the hormonal parameters TSH or thyroxine. This favors an immunological mechanism of pain association over hormonal [77].

Spinal degenerative disc disease (DDD), a form of OA, is associated with older age, elevated BMI, certain viral infections, genetics, mechanical loading, atherosclerosis, smoking, and nutrition. A cross-sectional analysis of over 4300 patients found those with AITD were 1.5 times more likely to have spinal DDD than those without [71]. The association persisted after adjusting for covariates, stratifying by BMI and TSH, and after excluding patients with known ACTD and spondylarthritis. Interestingly, the association also persisted among euthyroid patients with ATA, who were 1.7 times more likely to have spinal DDD. Elevated TPOAbs (> 5 IU/mL) were associated with having spinal DDD (OR 1.3) [71]. The fact that AITD was shown in this study to be associated with a non-erosive OA may indicate autoimmune mechanism causes that adversely impact cartilage biology. Since RA per se is associated with vertebral C1–C2 instability, the presence of more diffuse spinal involvement should call attention to potential overlap with AITD. The care of such patients might therefore require a multidisciplinary approach involving rheumatology, orthopedics, rehabilitation medicine, pain medicine, and neurology among others.

A large-scale retrospective cohort study revealed that RA patients had an adjusted HR of 2.75 (*p* < 0.001) of developing OA at multiple sites as compared to a propensity score matched non-RA control group over an observation period of 10 years [78]. The most common specified site for OA was the lower leg.

## AITD in RA and chronic widespread pain or fibromyalgia

Chronic widespread pain and fibromyalgia are increasingly reported as being independently associated with AITD [70, 79, 80]. Fibromyalgia has also been shown to have an increased association with RA. A study of RA patients from Australian rheumatology clinics found 42% had fibromyalgia using the 2011 American College of Rheumatology (ACR) classification criteria [81]. The patients with fibromyalgia had worse outcomes in all domains of health compared to RA patients without. A cross-sectional study found that concomitant fibromyalgia was independently associated with difficult-to-treat RA [82].

Fibromyalgia has been highly associated with AITD in multiple studies [15] (Table 2). In one such study, the close association of AITD with fibromyalgia in RA was investigated in a retrospective cohort [83]. Specifically, TPOAb but not TgAb was found to correlate with the presence of fibromyalgia in RA with an OR of 4.6 (*p* < 0.001). The finding is important because the evaluation of ATA in the initial assessment of patients with RA could offer significant prognostic and clinical insights into their likelihood of developing fibromyalgia or chronic widespread pain. Early detection of fibromyalgia in patients allows for appropriate interventions and support. This may include incorporating pain management strategies tailored to fibromyalgia, addressing psychological well-being through the management of depression and anxiety, and providing education and resources for coping with these medical conditions.

**Table 2:** Musculoskeletal manifestations of autoimmune thyroid disease vs rheumatoid arthritis

Musculoskeletal manifestation	Presentation in AITD	Presentation in RA	References
Inflammatory arthritis	Non-erosive arthritis that may involve DIPs	Typically erosive arthritis that spares the DIPs	Valderrama-Hinds [47]
Osteoarthritis	Peripheral OA with classic Heberden’s and Bouchard’s nodes, knee OA that can involve the patellofemoral joints	Secondary degenerative changes in joints classically affected by rheumatoid arthritis including the MCPs, PIPs, and wrists	Addamanta [74]
Spinal involvement	Affects the mobile parts of the spine in particular cervical and lumbar with disc degeneration and secondary facet arthritis	Classically involves the atlanto-dens articulation (C1–C2)	Shrestha [71]
Fibromyalgia and chronic widespread pain	Can present without signs of generalized inflammation	Presents as secondary fibromyalgia which improves with control of RA disease activity	Ahmad [83], Sheth [15]
Chondrocalcinosis	Associated with TPOAb	No direct association with rheumatoid arthritis	Tagoe [72]
Myopathy	More common in the presence of hypothyroidism	Not a feature of RA alone	Villar [84]
Other findings	Carpal tunnel syndrome, trigger finger, tendonitis, and capsulitis are reported in higher frequency	Carpal tunnel syndrome, trigger finger, tendonitis, and capsulitis are reported in higher frequency	Cakir [85]

## Other associations of the interaction of AITD and RA: female fertility and depression

### Fertility

Infertility is defined as an inability to achieve pregnancy after 1 year of regular intercourse without the use of contraceptives and subfertility is a delay in achieving pregnancy. Both RA and AITD affect fertility. RA is characterized by subfertility, for which there are myriad reasons [86]. Importantly, the ovulatory score has been shown to be significantly lower among RA patients, indicating they were less likely to ovulate [87]. The subfertility may be related to inflammation as well as medications used to manage RA [88]. Nonsteroidal anti-inflammatory drugs (NSAIDs) reversibly decrease fertility [89]. NSAIDs inhibit the cyclooxygenase 2 (COX-2) enzyme, thereby decreasing prostaglandin synthesis. COX-2 is active in the ovaries during follicular development [90]. Therefore, NSAID use is associated with anovulation (luteinized unruptured follicle syndrome) and may impact fertilization and implantation as well [89]. Corticosteroids, taken exogenously or produced in response to stress, can also cause infertility via actions on the hypothalamic-pituitary-gonadal axis [91]. Corticosteroids can decrease the synthesis of gonadotropin-releasing hormone (GnRH), luteinizing hormone, and follicle-stimulating hormone (FSH) and can bind the glucocorticoid receptor in the ovaries and testes, impairing reproduction directly [91]. Cyclophosphamide, an alkylating agent, may deplete ovarian reserve and stimulate ovarian failure [86]. Methotrexate is used not only for RA but also for terminating ectopic and intrauterine pregnancies. Methotrexate can cross the placenta and cause miscarriages during the first trimester of pregnancy [86]. Beyond biological causes of subfertility, RA patients may elect for smaller family sizes, try to conceive at an older age, and face limitations in sexual function [88]. According to a study of 830 patients with RA, approximately 30% felt their health status significantly affected their sexual activity, which was attributed to fatigue, mental distress, functional limitations, and lower self-efficacy [92].

Normal functioning of the thyroid is essential for reproduction. Alterations in thyroid hormones are associated with impaired folliculogenesis, spermatogenesis, fertilization, placental development, and fetal growth [93, 94]. In fact, screening for thyroid dysfunction is part of the initial infertility work-up [95]. There is evidence of TSH and thyroid hormone receptors on ovarian surface epithelium and oocytes as well as deiodinases in the endometrium [93]. T3 combined with FSH promotes granulosa cell proliferation and upregulates expressions of several factors in early placental extravillous trophoblasts including matrix metalloproteinases 2, 3, and fetal fibronectin [93]. Insufficient thyroid hormone production can lead to hyperprolactinemia [86]. Hyperprolactinemia impairs the pulsatile release of GnRH, suppresses LH levels, and causes ovulatory dysfunction [86].

In addition to thyroid hormones, thyroid autoantibodies can affect reproduction. TPOAb is associated with impaired embryogenesis and lower fertilization rates. It is possible that the ATA themselves affect fertility by targeting oocyte antigens and creating a hostile immune and cytotoxic environment for the maturing oocytes [95]. Alternatively, the subfertility may be caused by associated thyroid hormone deficiency or general autoimmunity [93].

Both overt hypo- and hyperthyroidism as well as AITD in general are risk factors for preterm birth. A meta-analysis of over 47,000 participants found TPOAb-positive women had 1.3 times the odds of having a preterm birth (< 37 weeks’ gestational age) and 2.5 times the odds of having a very preterm birth (< 32 weeks’ gestational age) compared to TPOAb-negative women. Furthermore, a sensitivity analysis showed that the association of subclinical hypothyroidism and preterm birth disappeared when adjusting for TPO-antibody positivity, indicating that the ATA may underly this association [94].

### Depression

AITD and RA are associated with mental health disorders. A meta-analysis including over 44,000 patients showed those with AITD, HT, or subclinical or overt hypothyroidism scored three times higher on standardized depression instruments [96]. The same group was found to have two times the odds of having an anxiety disorder [96]. A retrospective case study of 93 inpatients in a mood disorders unit found that patients with treatment-resistant depression were significantly more likely to have subclinical or overt hypothyroidism than those with non-treatment-resistant depression (22% vs. 2%, *p* < 0.01) [97]. Wu et al. [98] studied outcomes in non-autoimmune hypothyroidism and AITD groups among 1718 outpatients with treatment-naïve major depressive disorder (MDD). Although both groups were associated with the duration of MDD, Hamilton Depression Scale score, severity of anxiety, and MS, only the AITD group with subclinical hypothyroidism was associated with suicide attempts [98].

Depression also has a significant association with RA. A meta-analysis that included over 13,000 patients found the prevalence of MDD to be 17% among RA patients versus 5% globally [99, 100]. The comorbidity of RA and depression may be due to shared pathophysiology. Certain cytokines are elevated in persons with depression as well as RA, including IL-1β, IL-6, and TNF-α. These peripheral cytokines may be implicated in depression by activating the blood-brain barrier endothelium, being transported across the blood-brain barrier into the central nervous system (CNS), (primarily TNF-α) and via circumventricular organs. The inflammatory reflex, whereby the vagus nerve facilitates communication about peripheral cytokines to the CNS, which can then regulate peripheral inflammation and cytokine production may further implicate cytokines in depression. Neuroimaging studies have demonstrated changes in the brain that are associated with both systemic inflammation seen in RA and mood changes. For instance, inflammation can activate the insula, and insula activation is associated with subjective fatigue. In addition, systemic inflammation is associated with decreased functional connectivity in the corticostriatal reward circuitry which is seen in depression. Also, the pain and fatigue that are common in RA may cause or worsen depression. Indeed, IL-6 has been shown to have a role in the production of pain and fatigue in RA and may also have a role in depression [99].

## Conclusion

The intersection between RA and AITD is intricate. Although the latter has been found in increased frequency in the former, the true level of overlap may be even higher than documented since AITD diagnosed using more comprehensive criteria including ultrasound and histology is more frequent than with the use of ATA for case identification [101]. We have presented evidence in this review for interactions between RA and AITD beyond just clinical overlap, including possible synergism in their effects on CVD, cartilage, joints, pain, fatigue, mood, and effects on general well-being and fertility. The question of why the thyroid gland is such a common target of autoimmune involvement is complex [102]. Is the thyroid gland an important source of antigenic determinants of autoimmunity, or is it involved in the miseducation of lymphocytes that could influence the breakdown of immune regulation? We believe, given the strength of emerging data, that the thyroid can no longer be ignored in our understanding of systemic ACTD and autoimmunity in general. Future studies should be directed at investigating AITD, its potential pathways of tissue injury, and whether mitigating thyroid autoimmunity could help reduce the disease burden of the autoimmune overlap syndromes including RA.

## Data Availability

The data supporting the study findings are available within the article.
